# A phase I study of the lipophilic thymidylate synthase inhibitor Thymitaq™ (nolatrexed dihydrochloride) given by 10-day oral administration

**DOI:** 10.1038/sj.bjc.6690146

**Published:** 1999-02

**Authors:** D I Jodrell, A Bowman, R Rye, B Byrne, A Boddy, I Rafi, G A Taylor, A Johnston, N J Clendeninn

**Affiliations:** 1ICRF Medical Oncology Unit, Western General Hospital, Edinburgh EH4 2XU, UK; Cancer Research Unit, University of Newcastle, Framlington Place, Newcastle upon Tyne, UK; Agouron Pharmaceuticals, 10350 N. Torrey Pines Road, La Jolla, CA, 92037-1020, USA

**Keywords:** nolatrexed, oral, 10-day administration, phase I

## Abstract

2-Amino-3,4-dihydro-6-methyl-4-oxo-5-(4-pyridylthio)-quinazoline dihydrochloride (nolatrexed dihydrochloride, Thymitaq, AG337), a specific inhibitor of thymidylate synthase, was developed using protein structure-based drug design. Intravenously administered nolatrexed is active clinically. As oral bioavailability is high (70–100%), nolatrexed was administered orally, 6 hourly for 10 days, at 3-week intervals, and dose escalated from 80 to 572 mg m^−2^day^−1^in 23 patients. Common toxicity criteria (CTC) grade 3 toxicities included nausea, vomiting, stomatitis and liver function test (LFT) abnormalities. Thrombocytopenia (grade 1 or 2) occurred at doses ≥ 318 mg m^−2^day^−1^and neutropenia (grade 2) at 429 and 572 mg m^−2^day^−1^. An erythematous maculopapular rash occurred at dosages ≥ 318 mg m^−2^day^−1^(7 out of 19 patients). LFT abnormalities occurred in two out of six patients (grade 3 or 4 bilirubin and grade 3 alanine transaminase) at 572 mg m^−2^day^−1^. Nolatrexed plasma concentrations 1 h after dosing were 6–16 μg ml^−1^, and trough 3–8 μg ml^−1^, at 572 mg m^−2^day^−1^. Inhibition of thymidylate synthase was demonstrated by elevation of plasma deoxyuridine. Six-hourly oral nolatrexed for 10 days was associated with antiproliferative effects, but nausea and vomiting was dose limiting at 572 mg m^−2^day^−1^. Nine patients were treated at 429 mg m^−2^day^−1^; three out of nine experienced grade 3 nausea, but 17 out of 22 treatment courses were completed (with the co-administration of prophylactic antiemetics) and this dose level could be considered for phase II testing. *1999 Cancer Research Campaign*


British Journal of Cancer (1999) 79(5/6), 915–920
© 1999 Cancer Research Campaign
Article no. bjoc.1998.0146

### A phase I study of the lipophilic thymidylate synthase inhibitor Thymitaq™ (nolatrexed dihydrochloride) given by 10-day oral administration

**DI Jodrell[1], A Bowman[1], R Rye[1], B Byrne[1], A Boddy[2], I Rafi[2], GA Taylor[2], A Johnston[3] and NJ Clendeninn[3]**

[1]ICRF Medical Oncology Unit, Western General Hospital, Edinburgh EH4 2XU, UK; [2]Cancer Research Unit, University of Newcastle, Framlington Place, Newcastle upon Tyne, UK; [3]Agouron Pharmaceuticals, 10350 N. Torrey Pines Road, La Jolla, CA 92037-1020, USA

**Summary** 2-Amino-3,4-dihydro-6-methyl-4-oxo-5-(4-pyridylthio)-quinazoline dihydrochloride (nolatrexed dihydrochloride, Thymitaq, AG337), a specific inhibitor of thymidylate synthase, was developed using protein structure-based drug design. Intravenously administered nolatrexed is active clinically. As oral bioavailability is high (70–100%), nolatrexed was administered orally, 6 hourly for 10 days, at 3-week intervals, and dose escalated from 80 to 572 mg $m^{-2}$ $day^{-1}$ in 23 patients. Common toxicity criteria (CTC) grade 3 toxicities included nausea, vomiting, stomatitis and liver function test (LFT) abnormalities. Thrombocytopenia (grade 1 or 2) occurred at doses ≥ 318 mg $m^{-2}$ $day^{-1}$ and neutropenia (grade 2) at 429 and 572 mg $m^{-2}$ $day^{-1}$. An erythematous maculopapular rash occurred at dosages ≥ 318 mg $m^{-2}$ $day^{-1}$ (7 out of 19 patients). LFT abnormalities occurred in two out of six patients (grade 3 or 4 bilirubin and grade 3 alanine transaminase) at 572 mg $m^{-2}$ $day^{-1}$. Nolatrexed plasma concentrations 1 h after dosing were 6–16 μg $ml^{-1}$, and trough 3–8 μg $ml^{-1}$, at 572 mg $m^{-2}$ $day^{-1}$. Inhibition of thymidylate synthase was demonstrated by elevation of plasma deoxyuridine. Six-hourly oral nolatrexed for 10 days was associated with antiproliferative effects, but nausea and vomiting was dose limiting at 572 mg $m^{-2}$ $day^{-1}$. Nine patients were treated at 429 mg $m^{-2}$ $day^{-1}$; three out of nine experienced grade 3 nausea, but 17 out of 22 treatment courses were completed (with the co-administration of prophylactic antiemetics) and this dose level could be considered for phase II testing.

**Keywords:** nolatrexed; oral; 10-day administration; phase I

Thymidylate synthase (TS) is the rate-limiting enzyme essential for the de novo synthesis of the pyrimidine nucleotide thymidylate. Because thymine bases are only found in DNA, specific inhibitors of thymidylate synthase may be expected to induce an antiproliferative effect which is uncomplicated by effects at other biochemical loci, i.e. protein synthesis. It might be expected that such an agent would be relatively free from side-effects on non-proliferating tissues.

A number of drugs which are known to inhibit thymidylate synthase have been synthesized and used clinically. The most widely used is 5-fluorouracil (5-FU) (Valeriote and Santelli, 1984), a substrate analogue which is rapidly metabolized within the cell to a number of fluorinated nucleotide moieties. One of these metabolites, 5-fluorodeoxyuridine monophosphate (5-FdUMP) is a potent inhibitor of thymidylate synthase (Santi et al, 1974). However, 5-FU has multiple biochemical sites of action because other nucleotide metabolites may be incorporated into DNA or RNA (Pinedo and Peters, 1988).

Therefore, a selective inhibitor of thymidylate synthase may be more effective than 5-FU. In particular, such an inhibitor would be more likely to have a unique locus of action, would not be incorporated into nucleic acids and might not be susceptible to catabolic degradation. A number of folate-based thymidylate synthase inhibitors have been synthesized and entered clinical trial. The first of these, CB 3717, displayed promising clinical activity (Calvert et al, 1986, 1987; Bassendine et al, 1987; Cantwell et al, 1988), but its development was abandoned because of sporadic and unpredictable nephrotoxicity and myelotoxicity. A successor to CB 3717, raltitrexed (Tomudex™) (Jackman et al, 1991), has been shown to be non-nephrotoxic (Jodrell et al, 1991) and is currently in use clinically in the treatment of patients with metastatic colorectal cancer.

Both CB 3717 and Tomudex™ are 'classical antifolates' in that they enter the cell via the 'reduced folate carrier' protein and contain a terminal glutamate moiety which renders them substrates for the folyl-polyglutamate synthetase enzyme (FPGS). Metabolism to intracellular polyglutamate forms by FPGS greatly increases the potency of these drugs and also leads to intracellular retention. However, variations in the rate of formation and retention of these polyglutamates may well be responsible for the variable toxicity observed with these drugs. Further, the possession of a glutamate residue renders the drug hydrophilic and precludes its entry into tissues which do not possess a folate transport system. In particular, classical antifolates penetrate the central nervous system poorly such that intrathecal or high-dose administration is necessary.

Nolatrexed dihydrochloride (Thymitaq™, AG337) (Jackson et al, 1992; US Patent Application, 1992) is a lipophilic inhibitor of thymidylate synthase which should circumvent the problems with classical antifolates outlined above. It was designed by Agouron Pharmaceuticals using X-ray crystallographic techniques and molecular modelling (Appelt et al, 1991), and is the first potent lipophilic thymidylate synthase inhibitor to be proposed for clinical study.

*Received 6 April 1998*
*Revised 27 May 1998*
*Accepted 22 June 1998*

*Correspondence to:* D Jodrell, ICRF Medical Oncology Unit, Western General Hospital, Edinburgh EH4 2XU, UK

Nolatrexed entered clinical trial at Newcastle General Hospital under the auspices of the CRC Phase I/II Clinical Trials Committee in November 1992. Using a continuous 24-h infusion, the maximum dose administered was 1073 mg m$^{-2}$ and no systemic toxicity was encountered, but CTC grade 2 local toxicity (phlebitis) was observed (Rafi et al, 1995). This local toxicity necessitated the use of central venous catheters (Hickman lines). Preclinical data had suggested that nolatrexed would be more effective when given as a prolonged infusion and, therefore, studies to investigate the administration of nolatrexed when given as 5- and 10-day infusions have also been performed (Rafi et al, 1998; Creaven et al, 1998).

Prolonged intravenous administration requires either prolonged hospital admission or the use of ambulatory infusion devices. However, nolatrexed also demonstrated significant oral bioavailability in two species. In rats, fasted before treatment, the oral bioavailability of nolatrexed at doses of 20, 40 and 80 mg kg$^{-1}$ was 53%, 50% and 30% respectively. These doses equate to doses of 120, 240 and 480 mg m$^{-2}$. In dogs (fasted overnight), nolatrexed delivered in 5% dextrose by gavage was > 90% bioavailable. The bioavailability of the proposed clinical formulation (nolatrexed in capsules with microcrystalline cellulose and crospovidone) was 70% and 107% in one dog and 87% in a second dog, when dosing was preceded and followed by water gavage. In patient studies of 5-day oral administration, bioavailability was high (70–100%) (Rafi et al, 1996). To study the feasibility and effects of more prolonged oral administration, a phase I study of 10-day administration was undertaken.

The objectives of the study were: (a) to determine the maximum tolerated dose of nolatrexed when given orally to cancer patients in divided doses over 10 days; (b) to establish the dose-limiting and other toxicities associated with the repeated oral administration of nolatrexed; (c) to study the pharmacokinetics of nolatrexed in individual patients using a limited number of plasma samples; (d) to determine whether the plasma concentrations achieved cause inhibition of the target enzyme, thymidylate synthase; (e) to document any anti-tumour effects related to treatment with nolatrexed; and (f) to propose a suitable dose for the phase II evaluation of activity.

### PATIENTS AND METHODS

This was a non-randomized ‘phase I’ study with dose escalation. The methodology used followed the guidelines set out in the CRC Phase I/II Committee Operation manual. The study was conducted in accordance with the Declaration of Helsinki and ethical approval was granted by the Lothian Medicine and Clinical Oncology Research Ethics Sub-Committee (reference: 1702/94/4/70).

The following criteria were used to assess eligibility: a histologically proven diagnosis of a malignant disease for which no satisfactory treatment existed or against which established treatments had failed; patients had to be capable of understanding the nature of the trial and must have been capable of giving written informed consent; WHO performance status of 0–2. Patients had not received previous chemotherapy for 4 weeks and had recovered fully from previous myelosuppressive chemotherapy. No radiotherapy, nitrosoureas or mitomycin C were allowed within the preceding 6 weeks. Evidence of adequate haematological reserve and renal and hepatic function were required [haemaglobin (Hb) ≥ 10 g dl$^{-1}$, white blood cells (WBC) ≥ 4.0 × 10$^{9}$l$^{-1}$, platelets ≥ 100 × 10$^{9}$ l$^{-1}$], normal renal function (calculated creatinine clearance ≥ 60 ml min$^{-1}$) and bilirubin.

**Table 1** Number of patients treated at each dose level

| Dose level | No. of patients | No. of cycles | No. of incomplete cycles (reason given) |
|---|---|---|---|
| 80 mg m$^{-2}$ day$^{-1}$ | 1 | 2 | 1 (PD) |
| 159 mg m$^{-2}$ day$^{-1}$ | 3 | 7 | 3 (PD) |
| 318 mg m$^{-2}$ day$^{-1}$ | 5[a] | 16 | 2 (toxicity) |
| 429 mg m$^{-2}$ day$^{-1}$ | 9[a] | 22 | 5 (4 toxicity 1 UT1) |
| 572 mg m$^{-2}$ day$^{-1}$ | 6 | 8 | 5 (DLT defined) |

[a]One patient had two cycles at 318 mg m$^{-2}$ day$^{-1}$ and three cycles at 429 mg m$^{-2}$ day$^{-1}$.

Other liver function tests (e.g. alkaline phosphatase, alanine transaminase, γ-glutamyl transpeptidase) were required to be less than twice the upper limit of normal, unless clearly due to the presence of tumour. A life expectancy of at least 3 months was expected and all patients were ≥ 18 years old.

Patients were excluded in the event of: brain metastases or primary brain tumours which precluded obtaining informed consent or were likely to require treatment with radiotherapy, severe pre-existing medical conditions, evidence of bone marrow involvement with tumour, concurrent use of other experimental agents or anti-cancer therapy, haematological malignancies, the need for concurrent administration of trimethoprim, antiepileptics, cotrimoxazole or pyrimethamine. Other exclusion criteria included women who were pregnant or breast feeding (patients utilized medically approved contraceptive precautions, if necessary, during the trial and for 4 weeks afterwards), patients who had received very extensive previous therapy in whom bone marrow function was likely to be compromised, patients receiving folate-containing vitamin supplements.

### Drug administration and dose escalation

Drug was supplied in size 4 and size 2 capsules containing 20 mg and 80 mg of nolatrexed (as free base) respectively. The inactive contents of the capsules comprised microcrystalline cellulose, crospovidone (5% weight of filled capsule) and magnesium stearate (0.5% weight of filled capsule). The capsules were stored in a cool, dry place (not refrigerated). Doses were to be administered at least 30 min before or 2 h after food. All nolatrexed doses and concentrations are expressed as free base.

The planned starting dose of nolatrexed was 40 mg m$^{-2}$ administered every 6 h; i.e. total daily dose = 159 mg m$^{-2}$ and total dose = 1590 mg m$^{-2}$. Total daily dose was calculated and divided by four to give the 6-hourly dose. The 6-hourly dose was rounded up or down to the nearest 20 mg in view of the available capsule sizes. Ten days of nolatrexed constituted one course of treatment. Treatment courses were repeated every 21 days, provided any drug-related non-haematological toxicities had resolved and WBC ≥ 3.0 × 10$^{9}$ l$^{-1}$ and platelets ≥ 100 × 10$^{9}$ l$^{-1}$.

Dose escalation was guided by the incidence of drug-related toxicity, and in the absence of drug-related toxicity the dose was doubled. Doses could be escalated within individual patients if no drug-related toxicities were observed at the previous dose level. Three patients were entered per dose level if no drug-related toxicities ≥ common toxicity criteria (CTC) grade 2 (except nausea and vomiting) were observed. Five patients per dose level were to be

 *© Cancer Research Campaign 1999*

**Table 2** Toxicities possibly or probably related to nolatrexed

**A** Nausea

| Daily dose (mg m$^{-2}$) | Number of patients at each CTC grade | | | | |
|---|---|---|---|---|---|
| | 0 | 1 | 2 | 3 | 4 |
| 80 | | | | 1 | |
| 159 | 1 | 2 | | | |
| 318 | 1 | 1 | 1 | 2 | |
| 429 | 1 | 3 | 2 | 3 | |
| 572 | | | | 6 | |

**B** Bilirubin

| Daily dose (mg m$^{-2}$) | Number of patients at each CTC grade | | | | |
|---|---|---|---|---|---|
| | 0 | 1 | 2 | 3 | 4 |
| 80 | | | 1 | | |
| 159 | | | 2 | 1 | |
| 318 | 3 | | | 2 | |
| 429 | 8 | | | 1 | |
| 572 | 4 | | | 1 | 1 |

entered if ≥ CTC grade 2 toxicities were observed. Additional patients were to be entered at the recommended phase II dose.

The maximum tolerated dose was defined as the dose associated with an incidence of dose-limiting toxicity (DLT) in three or more of the five patients per dose level. In this study, a DLT was defined as grade III or IV myelosuppression of more than 4 days duration and/or associated with neutropenic sepsis. Nausea (and/or vomiting) was considered dose limiting if it was uncontrolled by antiemetic therapy and/or limited the completion of the prescribed course of treatment. For other non-haematological toxicities, DLT was defined as the incidence of grade III or IV toxicity (excluding alopecia).

Before treatment and before each course, patients underwent clinical examination, full blood count, urea and electrolytes, liver function tests, calculated creatinine clearance (or chromium EDTA clearance if borderline renal function). During the first course of treatment, full blood count, urea and electrolytes and liver function tests were performed twice weekly and weekly thereafter.

### Pharmacokinetic and pharmacodynamic sampling and assessment

Limited pharmacokinetic sampling was performed during course 1. Heparinized blood samples (3 ml) were collected, for subsequent plasma drug assay, before drug administration, and 30–60 min after dosing on days 1, 4, 8 and 10. Plasma and deoxyuridine (dUrd) concentrations were also assessed during course 1 on days 1 (pretreatment) and 10. The concentration of nolatrexed was determined in each plasma sample using a high-performance liquid chromatography (HPLC) method with UV quantification and a semiautomated method was used for the determination of dUrd concentrations in plasma, both of which have been described previously (Rafi et al, 1995).

Patients with assessable disease had such clinical measurements and scans performed as were necessary to document the status of their disease. Clinical measurements were performed within the week before the patient going on study, and scans were performed within the month before the patient going on study. Assessable disease was reassessed after three cycles of treatment, unless there was clinical/biochemical evidence of disease progression. Treatment was continued until there was evidence of disease progression or unacceptable toxicity.

### RESULTS

### Patient characteristics and toxicities

Twenty-three patients were entered into the study (16 men and seven women). The median age was 57 (range 44–70). Median performance status was 1 (range 0–2). Colorectal cancer was the most common tumour type (12 patients) and other tumour types were lung [non-small-cell lung cancer (NSCLC); four patients] gastric (two); mesothelioma (one); renal (one); head and neck (squamous) (one); unknown primary (adenocarcinoma) (two). The majority of patients had had prior surgery (20) and chemotherapy (17). Seven patients had received prior radiotherapy and one patient endocrine treatment.

In view of concerns regarding the potential schedule dependency of toxicities related to other antimetabolites, one patient was treated at a preliminary dose level of 80 mg m$^{-2}$ day$^{-1}$. This patient had no evidence of drug-related antiproliferative toxicities, but subsequently developed progressive disease with bowel obstruction leading to nausea and vomiting. Therefore, three patients were accrued to the planned start dose, 159 mg m$^{-2}$ day$^{-1}$. The number of patients and courses at each of five dose levels is shown in Table 1. CTC grade III nausea and vomiting, which was probably or definitely related to nolatrexed and was associated with failure to complete the prescribed course of treatment, was encountered at 318 mg m$^{-2}$ day$^{-1}$ (Table 2A). This dose level was expanded to five patients and regular oral antiemetic prophylaxis (domperidone 20 mg every 6 h) was introduced. After completion of this dose level, three patients were entered at 429 mg m$^{-2}$ day$^{-1}$ and these patients were treated successfully with the co-administration of domperidone, as above.

At a daily dose of 572 mg m$^{-2}$ day$^{-1}$ (143 mg m$^{-2}$, every 6 h), nausea and vomiting became dose limiting, despite regular domperidone. All six patients entered developed persistent grade III nausea and five out of eight cycles were incomplete. This was felt to represent the dose-limiting toxicity and the maximum tolerated dose appeared to have been defined. In patients who ‘failed’ despite regular domperidone, oral ondansetron was used, and, in two patients, oral ondansetron (4–8 mg twice daily) was used as antiemetic prophylaxis. One patient was treated with a combination of oral ondansetron and dexamethasone (4 mg twice daily), but became agitated and discontinued therapy. In addition to nausea, two out of six patients developed grade III stomatitis at this dose level.

Therefore, the 429 mg m$^{-2}$ day$^{-1}$ dose level was expanded to gain additional experience at what was likely to represent the recommended phase II dose. Grade 3 nausea was noted in three out of nine patients and 5 out of 22 cycles were incomplete. Grade 2 stomatitis was encountered in two patients at 429 mg m$^{-2}$ day$^{-1}$, but grade 3 stomatitis was not reported at this dose level.

Myelosuppression (thrombocytopenia and/or neutropenia) was recorded at daily doses ≥ 318 mg m$^{-2}$. This did not exceed CTC grade 2 in any patient. At the maximum dose tested (572 mg m$^{-2}$ day$^{-1}$), two out of six patients experienced grade 2 neutropenia and one out of six experienced grade 2 thrombocytopenia.

A skin rash was noted in eight patients and appeared to be most prominent in sun-exposed areas. It was generally mild and self

© Cancer Research Campaign 1999

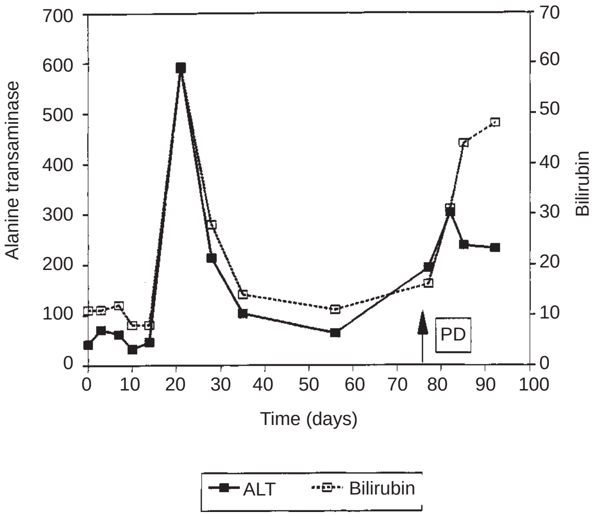

**Figure 1** Changes in liver function tests (alanine transaminase and bilirubin) after the administration of nolatrexed (day 0) in one patient. PD, disease progression

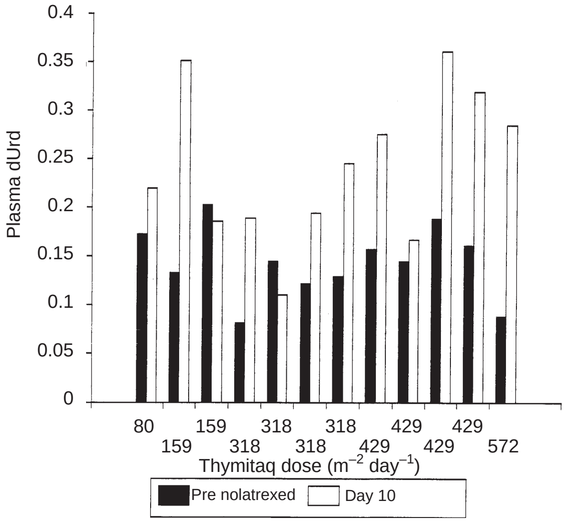

**Figure 2** Plasma deoxyuridine estimations before nolatrexed administration and on day 10 in 12 patients

**Table 3** Nolatrexed plasma concentrations. Patients sampled on days 1, 3 and 8. All doses and concentrations are expressed as nolatrexed-free base

| Dose (mg m$^{-2}$ day$^{-1}$) | Time point | Median (µg ml$^{-1}$) | Range (µg ml$^{-1}$) |
|---|---|---|---|
| 572 | 30–60 min | 12.3 | 5.8–16.2 |
| | Trough | 4.7 | 2.7–8.0 |
| 429 | 30–60 min | 7.4 | 3.0–11.2 |
| | Trough | 1.8 | 0.6–2.6 |
| 318 | 30–60 min | 8.1 | 4.7–8.3 |
| | Trough | 1.0 | 0.2–1.4 |
| 159 | 30–60 min | 2.2 | 1.9–2.8 |
| | Trough | 0.2 | 0.1–1.4 |

limiting, and therefore biopsies were not performed, although one patient treated at 572 mg m$^{-2}$ day$^{-1}$ was treated using dexamethasone and chlorpheniramine. Five additional patients also commented on increased sensitivity to sunlight, but no apparent rash.

Elevation of serum bilirubin was noted in nine patients (Table 2 B), with grade 3 or 4 toxicity occurring in 5 out of 20 patients treated at doses ≥ 318 mg m$^{-2}$ day$^{-1}$. These abnormalities were also associated with rises in serum alanine transaminase (ALT) with two out of six patients experiencing grade 3 toxicity at ≥ 572 mg m$^{-2}$ day$^{-1}$. In a population of patients with metastatic cancer, it was often difficult to assign causality, but in certain patients there was a clear temporal relationship to drug administration (Figure 1) and spontaneous resolution in the absence of any suggestion of disease progression. Data from these patients confirmed the relationship between drug administration and liver function test abnormalities which was self-limiting and not associated with any significant clinical sequelae.

### Nolatrexed pharmacokinetics

Pharmacokinetic data were available from 22 patients. Data were available from 23 courses of treatment because nolatrexed was administered at two successive dose levels in one patient. Nolatrexed was measured in plasma 30–60 min after capsule administration and at approximately 6 h (Table 3). There was no evidence of accumulation over the 10-day period of dosing. Median trough values, shown in Table 3, support the data of Rafi et al (1998), which demonstrated that nolatrexed clearance is dose dependent. Plasma concentrations of 3–11 µg ml$^{-1}$ were achieved in patients 30–60 min after dosing at the 429 mg m$^{-2}$ day$^{-1}$ dose level with 'trough' values of 0.6–2.6 µg ml$^{-1}$.

The 30–60 min concentration cannot be assumed to represent a true peak value and therefore cannot be used for correlation with the toxicities noted. When mean trough values were considered, no correlation could be found with the recorded toxicities.

### Plasma deoxyuridine (dUrd) concentrations

Data available from 12 out of 23 patients confirm that plasma dUrd concentrations rise (median rise = 90% of baseline) after oral administration of nolatrexed (Figure 2), as might be predicted by the achievement of relevant plasma concentrations of nolatrexed. The magnitude of the elevation of dUrd is similar to that seen after the intravenous administration of nolatrexed (Rafi et al, 1998). Data were only available in one out of six patients treated at the maximum dose because five out of six first cycles of treatment were not completed. Data were available from four out of nine patients receiving 429 mg m$^{-2}$ day$^{-1}$, and in three out of four the elevation in dUrd was > 75% compared with baseline. In this small number of patients, it was not possible to identify any relationships between change in dUrd and toxicity.

### Anti-tumour activity

Ten out of 23 patients had evaluable disease, and, of these, seven had progressive disease on treatment and three patients achieved disease stabilization; 122 days (adenocarcinoma small intestine, patient A), 219 days (gastric cancer, previous partial response with 5-FU, doxorubicin and mitomycin C, patient B) and 70 days (NSCLC), measured from the start of nolatrexed. Patient A received five cycles of nolatrexed and, on disease progression, was

© Cancer Research Campaign 1999

treated using 5-FU/folinic acid chemotherapy with further disease stabilization. He survived 23 months after treatment with nolatrexed. Patient B received six cycles of nolatrexed and remains alive 19 months after treatment.

### DISCUSSION

This paper describes a completed phase I trial of the novel TS inhibitor, nolatrexed, administered orally over 10 days, repeated at 3-weekly intervals. The maximum tolerated dose of nolatrexed administered using this schedule was 572 mg $m^{-2}$ $day^{-1}$ (143 mg $m^{-2}$ every 6 h). Nausea was the dose-limiting toxicity, causing discontinuation of therapy in five out of eight cycles on days 4–8 at 572 mg $m^{-2}$ $day^{-1}$, despite prophylactic antiemetic therapy including both dexamethasone and ondansetron. Nine patients were treated at the lower dose level of 429 mg $m^{-2}$ $day^{-1}$ and three out of nine patients experienced CTC grade 3 nausea. However, 17 out of 22 treatment courses were completed (with the co-administration of prophylactic antiemetics) and this dose level (429 mg $m^{-2}$ $day^{-1}$) could be considered for phase II testing of nolatrexed administered for 10 days by mouth.

In comparison, in the 10-day intravenous infusion study using nolatrexed reported by Creaven et al (1998), dose escalation to 716 mg $m^{-2}$ $day^{-1}$ was performed, but zero out of three patients completed this dose level and two out of three developed grade 4 myelosuppression. A dose level of 572 mg $m^{-2}$ $day^{-1}$ was tolerated with one out of four patients developing dose-limiting myelosupression, but concern was expressed at the high incidence of thrombotic complications using the continuous infusion protocol. When nolatrexed was administered by 5-day intravenous infusion (Rafi et al, 1998), the maximum tolerated dose was 904 mg $m^{-2}$ $day^{-1}$ and the recommended phase II dose was 800 mg $m^{-2}$ $day^{-1}$. The dose-limiting toxicity was neutropenia. Anti-tumour activity was noted with one patient with metastatic colorectal cancer achieving a partial response, lasting 3 months. Nausea and vomiting was reported as mild to moderate, although ondansetron was used in 9 out of 31 patients.

In our 10-day oral study, antiproliferative toxicities (neutropenia, thrombocytopenia and stomatitis) were documented at dose levels ≥ 318 mg $m^{-2}$ $day^{-1}$. CTC grade 1 thrombocytopenia (four out of nine patients), CTC grade 2 neutropenia (one out of nine patients) and CTC grade 2 stomatitis (two out of nine patients) were demonstrated at 429 mg $m^{-2}$ $day^{-1}$.

Liver function test (LFT) abnormalities were noted in a proportion of patients (CTC grade 3 ALT in 2 out of 19, CTC grade 3 bilirubin in 5 out of 19 and CTC grade 4 bilirubin in 1 out of 19) receiving doses ≥ 318 mg $m^{-2}$ $day^{-1}$. Of the six patients with elevation of their bilirubin levels, four had normal bilirubin levels at the start of treatment and two were marginally elevated (18 and 17 μmol $1^{-1}$; upper limit of normal, 16 μmol $1^{-1}$), but all six had normal ALT levels at the onset of therapy. These LFT abnormalities were self-limiting and there were no significant clinical sequelae. Transient LFT abnormalities are reported with raltitrexed, a TS inhibitor in regular clinical use [data sheet: raltitrexed (Zeneca)], and need not limit its usage.

Skin rashes were seen in eight patients and an additional five patients noticed apparent photosensitivity. The rashes were more prominent in sun-exposed areas. Skin toxicites are often identified in patients receiving antimetabolite drugs [data sheets: reltitrexed (Zeneca), methotrexate (Faulding), flurouracil (Faulding)].

A dose level of 429 mg $m^{-2}$ $day^{-1}$ might be considered appropriate for phase II testing, although prophylactic antiemetic therapy would be required. The availability of 20 and 80 mg capsules meant that patients were required to take three to five capsules every 6 h. In patients who are experiencing nausea which they associate with the treatment, this is a major undertaking and, therefore, capsules containing larger quantities of nolatrexed (50 and 200 mg) could be produced, and it would be appropriate to re-evaluate the higher doses tested in this study using any new formulation before proceeding to formal phase II testing. An alternative strategy would be the development of a sustained release formulation and this is also being considered.

The pharmacokinetic studies demonstrate that the drug is absorbed and plasma concentrations are achieved at 429 mg $m^{-2}$ $day^{-1}$ (≥ 0.8 μg $ml^{-1}$), which would be associated with anti-tumour activity in vitro (Webber et al, 1996). It is intended that the limited plasma concentration data collected in this study will be combined with data from studies using different schedules and routes of administration to develop a population pharmacokinetic model for nolatrexed. The plasma concentrations achieved in this study would be expected to result in inhibition of the target enzyme, thymidylate synthase, and the elevation of plasma deoxyuridine (dUrd) concentrations seen in these patients supports this.

In summary, it has been shown that nolatrexed can be administered every 6 h for 10 days at doses sufficient to cause antiproliferative toxicities and which show evidence of thymidylate synthase inhibition. The tolerable dose in this oral study is lower than the tolerable dose determined using 10-day continuous intravenous administration. The pharmaceutical preparation used in this oral study was associated with dose-limiting nausea and vomiting, which may be reduced by modification of the formulation and such studies are ongoing. Nolatrexed has been shown to be an active drug in the treatment of head and neck cancer and hepatocellular carcinoma (Clendeninn et al, 1996), and its clinical development has now progressed to phase III trials. The necessity for the administration of nolatrexed over a number of days and the thrombotic complications associated with 10-day intravenous infusions would suggest that oral administration would represent a significant advantage.

### ACKNOWLEDGEMENTS

The authors wish to acknowledge the support and advice of the CRC Phase I/II committee and the assistance of the CRC Data Centre in the preparation of this paper.

### REFERENCES

Appelt K, Bacquet RJ, Bartlett CA, Booth CL, Freer ST, Fuhry MA, Gehring MR, Herrmann SM, Howland EF and Janson CA (1991) Design of enzyme inhibitors using iterative protein crystallographic analysis. *J Med Chem* **34**: 1925–1934

Bassendine MF, Curtin NJ, Loose H, Harris AL and James OJW (1987) Induction of remission in hepatocellular carcinoma with a new thymidylate synthase inhibitor, CB3717. *J Hepatol* **4**: 349

Calvert AH, Alison DL, Harland SJ, Robinson BA, Jackman AL, Jones TR, Newell DR, Siddik ZH, Wiltshaw E and McElwain TJ (1986) A phase I evaluation of the quinazoline antifolate thymidylate synthase inhibitor, N10-propargyl-5,8-dideazafolic acid, CB3717. *J Clin Oncol* **4**: 1245–1252

Calvert AH, Newell DR, Jackman AL, Gumbrell LA, Sikora E, Grzelakowska-Sztabert B, Bishop JA, Judson IR, Harland SJ and Harrap KR (1987) Recent preclinical and clinical studies with the thymidylate synthase inhibitor N10-propargyl-5,8-dideazafolic acid (CB 3717). *NCI Monogr*: 213–218

Cantwell BM, Macaulay V, Harris AL, Kaye SB, Smith IE, Milsted RA and Calvert AH (1988) Phase II study of the antifolate N10-propargyl-5,8-dideazafolic acid (CB 3717) in advanced breast cancer. *Eur J Cancer Clin Oncol* **24**: 733–736

© Cancer Research Campaign 1999

Clendeninn NJ, Collier MA, Johnston AL, Loh KK, Cohn A, Kelly K, Glode LM, Stuart KE and Belani CP (1996) Thymitaq (AG337): a novel thymidylate synthase inhibitor with clinical activity in solid tumours. *Proc Am Assoc Cancer Res* **37**: 171

Creaven PJ, Pendyala L, Meopol NJ, Clendeninn NJ, Wu EY, Loewen GM, Proefrock A, Johnston A and Dixon M (1998) Initial clinical trial and pharmacokinetics of Thymitaq (AG337) by 10-day continuous infusion in patients with advanced solid tumors. *Cancer Chemother Pharmacol* **41**: 167–170

Jackman AL, Taylor GA, Gibson W, Kimbell R, Brown M, Calvert AH, Judson IR and Hughes LR (1991) ICI D1694, a quinazoline antifolate thymidylate synthase inhibitor that is a potent inhibitor of L1210 tumor cell growth in vitro and in vivo: a new agent for clinical study. *Cancer Res* **51**: 5579–5586

Jackson RC. Design and development of lipophilic inhibitors of thymidylate synthase. *Proc Am Assoc Cancer Res* **33**: 592–593

Jodrell DI, Newell DR, Morgan SE, Clinton S, Bensted JP, Hughes LR and Calvert AH (1991) The renal effects of N10-propargyl-5,8-dideazafolic acid (CB3717) and a non-nephrotoxic analogue ICI D1694, in mice. *Br J Cancer* **64**: 833–838

Pinedo HM and Peters GF (1988) Flurouracil: biochemistry and pharmacology. *J Clin Oncol* **6**: 1653–1664

Rafi I, Taylor GA, Calvete JA, Boddy AV, Balmanno K, Bailey N, Lind M, Calvert AH, Webber S, Jackson RC, Johnston A, Clendeninn N and Newell DR (1995) Clinical pharmacokinetic and pharmacodynamic studies with the non-classical antifolate thymidylate synthase inhibitor 2-amino-3,4-dihydro-6-methyl-4-oxo-5-(4-pyridylthio)-quinazoline dihydrochloride (AG337) given by 24 hour continuous intravenous infusion. *Clin Cancer Res* **1**: 1275–1284

Rafi I, Boddy AV, Taylor GA, Calvete JA, Bailey NB, Lind MJ, Newell DR, Calvert AH, Johnston A and Clendeninn NJ (1996) A phase I clinical study of the novel antifolate AG337 given by 5 day oral administration. *Proc Am Assoc Cancer Res* **37**: 178

Rafi I, Boddy AV, Calvete JA, Taylor GA, Newell DR, Bailey N, Lind MJ, Johnston A, Clendeninn N and Calvert AH (1998) Pre-clinical and phase I clinical studies with the non-classical antifolate thymidylate synthase inhibitor Thymitaq (AG337) given by prolonged administration. *J Clin Oncol* **16**: 1131–1141

Santi DV, McHenry CS and Sommer H (1974) Mechanism of interaction of thymidylate synthetase with 5-fluorodeoxyuridylate. *Biochemistry* **13**: 471–481

US Patent Application filed 31 March 1992

Valeriote F and Santelli G (1984) 5-Fluorouracil (FUra). *Pharmacol Ther* **24**: 107–132

Webber S, Bartlett CA, Boritzki TJ, Hillard JA, Howland EF, Johnston AL, Kosa M, Margosiak SA, Morse CA and Shetty BV (1996) AG337, a novel lipophilic thymidylate synthase inhibitor: in vitro and in vivo preclinical studies. *Cancer Chemother Pharmacol* **37**: 509–517

*© Cancer Research Campaign 1999*
